# Non-coding RNAs as potential therapeutic targets for receptor tyrosine kinase signaling in solid tumors: current status and future directions

**DOI:** 10.1186/s12935-023-03203-2

**Published:** 2024-01-10

**Authors:** Aysan Moeinafshar, Mohammad Nouri, Nima Shokrollahi, Mahdi Masrour, Amirmohammad Behnam, Sahand Tehrani Fateh, Hossein Sadeghi, Mohammad Miryounesi, Mohammad-Reza Ghasemi

**Affiliations:** 1 Center for Comprehensive Genetic Services, Shahid Beheshti University of Medical Sciences, Tehran, Iran; 2 School of Medicine, Tehran University of Medical Sciences, Tehran, Iran; 3 School of Medicine, Shahid Beheshti University of Medical Sciences, Tehran, Iran; 4 Center for Orthopedic Trans-Disciplinary Applied Research, Tehran University of Medical Sciences, Tehran, Iran; 5 Department of Medical Genetics, Faculty of Medicine, Shahid Beheshti University of Medical Sciences, Tehran, Iran

**Keywords:** Solid tumors, RTK-RNAs, Receptor tyrosine kinase, miRNA, lncRNA, circRNA

## Abstract

This review article presents an in-depth analysis of the current state of research on receptor tyrosine kinase regulatory non-coding RNAs (RTK-RNAs) in solid tumors. RTK-RNAs belong to a class of non-coding RNAs (nc-RNAs) responsible for regulating the expression and activity of receptor tyrosine kinases (RTKs), which play a critical role in cancer development and progression. The article explores the molecular mechanisms through which RTK-RNAs modulate RTK signaling pathways and highlights recent advancements in the field. This include the identification of potential new RTK-RNAs and development of therapeutic strategies targeting RTK-RNAs. While the review discusses promising results from a variety of studies, encompassing in vitro*, *in vivo, and clinical investigations, it is important to acknowledge the challenges and limitations associated with targeting RTK-RNAs for therapeutic applications. Further studies involving various cancer cell lines, animal models, and ultimately, patients are necessary to validate the efficacy of targeting RTK-RNAs. The specificity of ncRNAs in targeting cellular pathways grants them tremendous potential, but careful consideration is required to minimize off-target effects, the article additionally discusses the potential clinical applications of RTK-RNAs as biomarkers for cancer diagnosis, prognosis, and treatment. In essence, by providing a comprehensive overview of the current understanding of RTK-RNAs in solid tumors, this review emphasizes their potential as therapeutic targets for cancer while acknowledging the associated challenges and limitations.

## Background

Receptor tyrosine kinases (RTKs) are a diverse family of cell surface receptors that are crucial for normal cellular processes such as cell growth, differentiation, and survival [1]. These receptors are activated by binding to extracellular ligands, which then triggers a series of molecular events of intracellular signaling pathways that regulate gene expression and cellular behavior [2]. The initiation of this process commonly involves the dimerization of adjacent RTKs and subsequent autophosphorylation of tyrosine residues within the resulting dimer. This autophosphorylation event is triggered by the binding of an extracellular signaling ligand. Subsequently, a phosphorylation cascade is initiated within the downstream signaling pathway. Under normal circumstances, the activation of RTKs and their downstream pathways, as well as the binding of extracellular ligands, are tightly regulated. However, in pathological conditions like cancer, receptor tyrosine kinases RTKs can become constitutively activated in a ligand-independent manner [3]. Several crucial pathways, including epidermal growth factor receptor (EGFR), vascular endothelial growth factor receptor (VEGFR), platelet-derived growth factor receptor (PDGFR), stem cell tyrosine kinase receptor (c-Kit), c-Met, and insulin-like growth factor receptor (IGFR) signaling pathways, are particularly significant in this context [4–10]. These pathways are targeted by miRNAs, further emphasizing their importance in regulating aberrant RTK signaling.

**Fig. 1:**
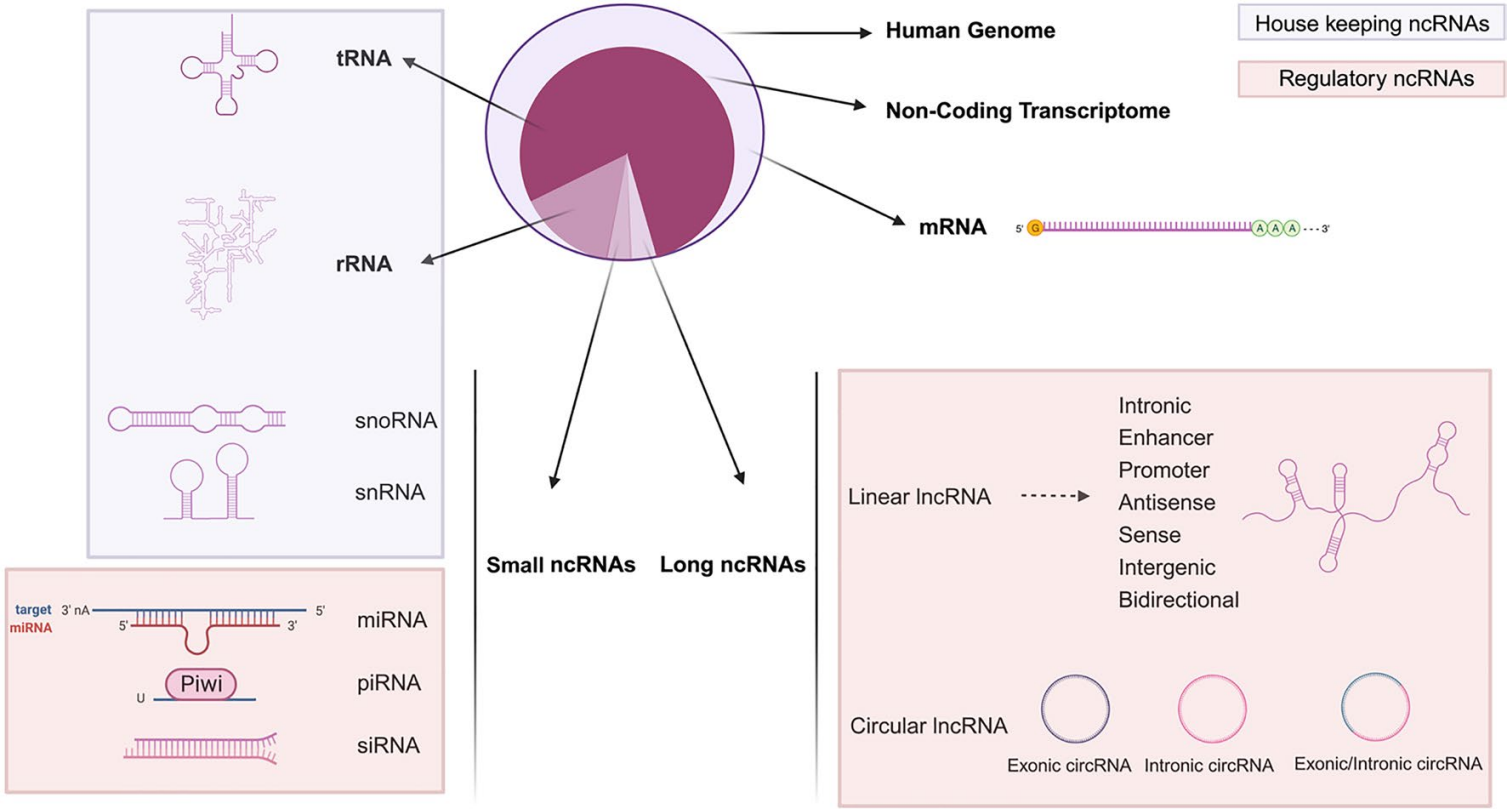
Classification of RNAs. RNAs can be classified into two major groups, coding RNA and non-coding RNAs. Traditionally, ncRNAs were categorized based on their size as small ncRNAs and long ncRNAs. However, a more recent classification system has emerged, which distinguishes between two main groups: housekeeping ncRNAs and regulatory ncRNAs

Dysregulation of RTK signaling, which is commonly observed in various types of cancer, has been implicated in tumor growth, invasion, and metastasis [11, 12]. To tightly control the expression and activity of RTKs, multiple mechanisms come into play, including post-translational modifications, protein–protein interactions, and regulation by non-coding RNAs (ncRNAs) [13]. ncRNAs, which do not code for proteins but play crucial roles in gene regulation are particularly important in this context. They can be classified into two major groups: housekeeping and regulatory ncRNAs. While the former classification was based on size, the newer categorization focuses on their importance in gene regulation (Fig. 1).

RTK-RNAs are a class of ncRNAs that specifically regulate the expression and activity of RTKs. These ncRNAs, mostly including microRNAs (miRNAs), piwi-interacting RNAs (piRNAs), long non-coding RNAs (lncRNAs), and circular RNAs (circRNAs) as a subtype of lncRNAs, have been shown to play important roles in the development and progression of solid tumors by regulating the expression of RTKs [14]. Depending on the specific RTK and RNA involved, RTK-RNAs can either activate or inhibit RTK signaling pathways [15]. For example, some RTK-RNAs may bind to and stabilize RTK mRNA, leading to increased expression and activation of the receptors. On the other hand, other RTK-RNAs can bind to and degrade RTK mRNA, resulting in decreased expression and activity of the receptors. Recent studies have identified several new RTK-RNAs that play important roles in cancer development and progression [16, 17]. These RNAs hold potential as targets for the development of novel cancer therapies. Additionally, therapeutic strategies targeting RTK-RNAs have shown promise in preclinical studies [18]. Overall, the regulation of RTK expression and activity by ncRNAs, specifically RTK-RNAs, is an important mechanism in cancer development and progression. Understanding the molecular mechanisms underlying the modulation of RTK signaling pathways by RTK-RNAs could lead to the development of new cancer therapies that target specific RTK-RNA interactions. Furthermore, the identification of RTK-RNAs as potential biomarkers for cancer diagnosis, prognosis, and treatment response could enable more personalized and effective cancer therapies.

This review article aims to provide a comprehensive overview of the potential therapeutic targets of ncRNAs in RTK signaling for solid tumors. The review specifically focuses on the different classes of ncRNAs, including miRNAs, tsRNAs, piRNAs, lncRNAs, and circRNAs, exploring their roles in cancer processes. The article then delves into the future prospects of ncRNA-based therapies targeting RTK signaling in solid tumors, emphasizing the potential to enhance cancer patient care. The growing understanding of the "dark matter" of the genome highlights the significant potential of targeting ncRNA signaling to impact cancer patient care. Throughout the review, the discussion centers on the current status and future directions of ncRNA-based therapies for RTK signaling in solid tumors.

## RTK-RNAs and their role in cancer

For a long time, health research primarily focused on studying the small portion of the genome responsible for coding proteins. It was believed that the remaining 98% had no significant function [19]. However, the ENCODE project revealed that this part of our genetic material is transcribed into numerous RNA molecules, known as non-coding RNAs (ncRNAs). These ncRNAs play a crucial role in regulating our body's functions and can be implicated in diseases, including cancer [20].

ncRNAs have emerged as key players in gene regulation and intercellular communication. They are involved in various processes such as coding, decoding, regulation, and expression of genes. Studies on ncRNA regulatory roles have demonstrated the existence of diverse ncRNA networks associated with different types of cancer. This breakthrough enables scientists to develop targeted strategies for cancer treatment and prevention by focusing on the specific ncRNAs encoded by our genes [21].

Deregulated expression of ncRNA has been directly linked to the development and progression of cancer, specifically affecting RTKs signaling pathways. RTKs are proteins on the cell surface that play a crucial role in cell proliferation and differentiation. Genetic alterations in ncRNA genes have also been associated with RTK-related cancers [22]. The different classes of ncRNAs can be broadly categorized based on their size including small ncRNAs such as miRNAs, siRNAs, and piRNAs along with lncRNAs, all of which contribute significantly to cancer biology.

While genetic variations in genes encoding ncRNAs have been linked to cancer, the number of identified cases is fewer compared to protein-coding genes. Additionally, the production or reduction of specific ncRNA molecules related to cancer can occur through different mechanisms, such as epigenetic, transcriptional, or post-transcriptional processes. Further research is needed to fully understand the precise control mechanisms of ncRNAs on genes and to explore ways to harness this knowledge for effective cancer treatment and prevention.

## Small non-coding RTK-RNAs

### The role of microRNAs in the RTK pathway in cancer

**Fig. 2:**
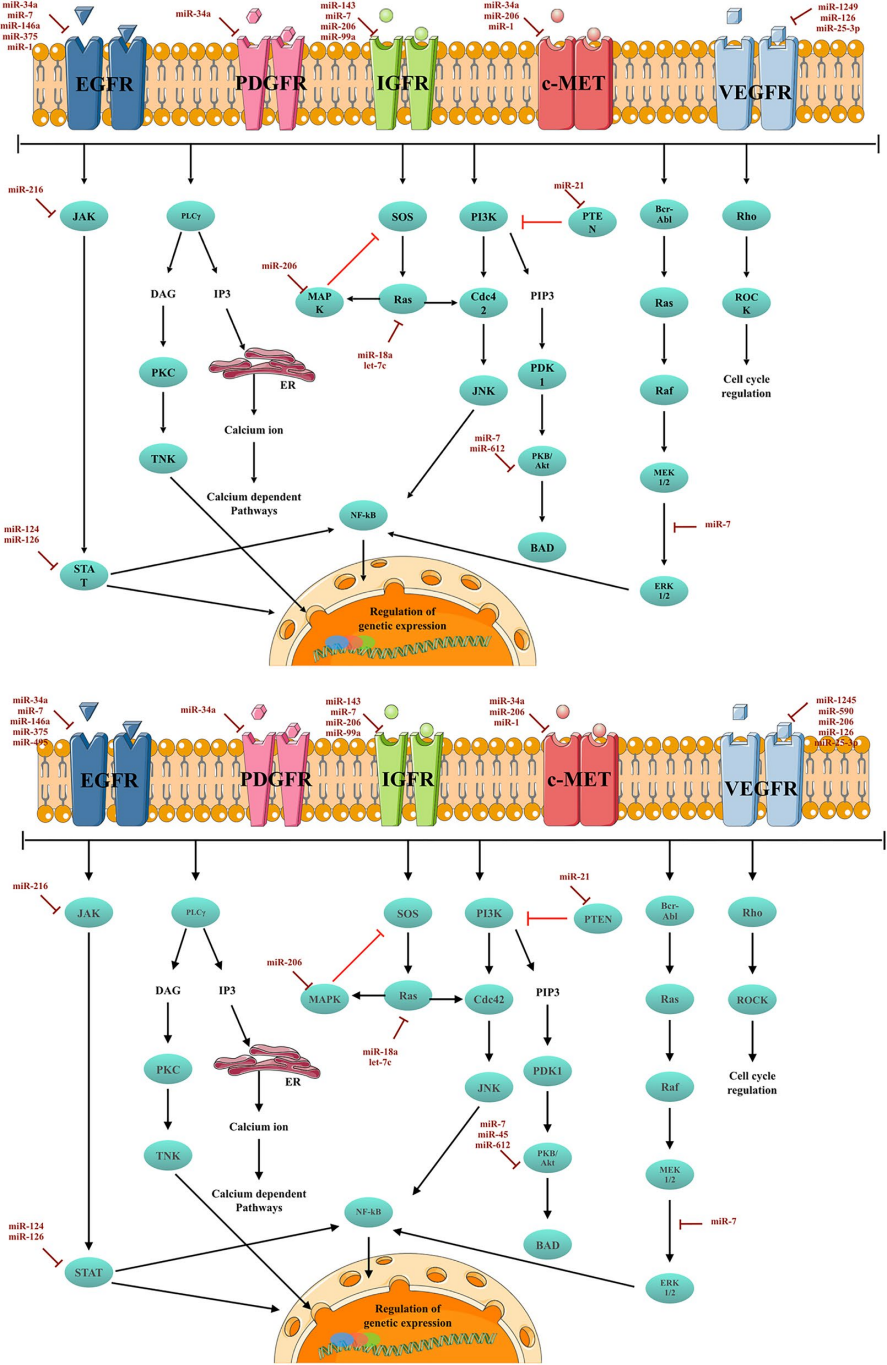
RTK-Mediated Signaling Pathways and Associated microRNAs in Solid Tumors This figure illustrates the signaling pathways mediated by receptor tyrosine kinases (RTKs) in solid tumors, along with the microRNAs that target different components of these pathways. The microRNAs have the ability to regulate the expression of both the RTK receptors and the downstream signaling cascades. The figure was created using the Servier Medical Art Commons Attribution 3.0 Unported License (http://smart.servier.com, accessed on 10 July 2023). Please note that the figure is for illustrative purposes only and may not depict the exact molecular interactions or signaling pathways in every solid tumor

MicroRNAs (miRNAs) are a type of regulatory ncRNAs that primarily function to regulate gene expression by interacting with recognition sites in the 3'-UTR region of mRNA, thereby affecting mRNA stability [23]. The expression of miRNA involves several post-transcriptional cleavage steps. Initially, the transcription of the miRNA locus results in the production of primary miRNA (pri-miRNA). The pri-miRNA then undergoes two cleavage steps, first by the microprocessor enzyme in the nucleus and then by the dicer enzyme in the cytoplasm. The first cleavage step generates pre-miRNA, which is subsequently transported into the cytoplasm by exportin-5 for the final cleavage step, resulting in the formation of mature miRNA. The mature miRNA is a double-stranded polyribonucleotide consisting of a guide strand and a passenger strand. Following the production of the mature form, the miRNA is attached to the argonaute protein (AGO), the passenger strand is removed, and the miRNA-induced silencing complex (miRISC) is formed, consisting of AGO and the guide strand. Any alterations in miRNA expression can impact the expression of target genes and disrupt cellular homeostasis, potentially leading to the development of various diseases, including cancer [24]. These alterations can arise from changes in components of the miRNA processing pathways (e.g., mutations in the dicer gene), genetic variations in miRNA-encoding loci, epigenetic regulation of miRNA expression, and silencing of miRNA expression by long non-coding RNAs (Fig. 2) [25–28].

Among the various genes that are targeted by miRNAs, RTKs play a crucial role in conjunction with microRNAs, particularly in stress signaling. This association is especially significant in disease-related pathological conditions such as cancer. miRNAs have the ability to function as both oncogenes and tumor suppressors by participating in downstream RTK signaling pathways and regulating the expression of RTK genes [29, 30]. A variety of microRNAs have been associated with the regulation of the signaling pathways downstream of the activated RTKs. For instance, miR-216 modulates the EMT process by modulating the JAK2/STAT3 pathway [31].

The actions and effects of RTKs mentioned above have led to the observation of alterations in their expression in various types of malignancies. One of the key factors contributing to these alterations is the investigation of changes in the levels of miRNAs that target RTKs. This area of research has received significant attention and has been extensively studied.

miR-34a is a microRNA that targets various factors associated with cancer. It regulates cellular processes such as cellular survival, stemness and differentiation, cell cycle, invasion, epithelial-mesenchymal transition (EMT), metabolism, immunity, and epigenetics. RTKs including c-Met, EGFR, AXL, PDGFR-B, and ErbB2 are among the targets of miR-34a [32]. Down-regulation of miR-34a has been reported in several malignancies like breast cancer, colon cancer, gastric cancer, non-small cell lung cancer, and prostate cancer [33–37]. On the other hand, up-regulation of miR-34a in both tissue and serum levels has been observed in medullary thyroid cancer, making it a potential biomarker for this malignancy [38, 39]. In breast cancer, miR-34a negatively impacts processes like vasculogenic mimicry, migration, and invasion by affecting the AXL receptor tyrosine kinase axis [40]. Overexpression of miR-34a suppresses tumor growth, invasion, and drug resistance in breast cancer [41]. In a meta-analysis conducted in 2017 by Imani et al., the use of miR-34a as a biomarker for breast cancer risk was examined. The analysis indicated a pooled sensitivity of 85.5% (OR 83.8–87% (95% CI)) and specificity of 70% (OR 65.8–74.1% (95% CI)). The overall area under the curve (AUC) was calculated to be 0.8 [42]. These findings indicate that miR-34a shows promise as a potential biomarker for breast cancer. Furthermore, the tumor suppressive effects of miR-34a suggest that it could be utilized as a treatment approach for breast cancer. Several studies have already been conducted on this matter [43–46]. These studies have explored the possibility of using an appropriate drug delivery system to target and deliver miR-34a specifically to breast cancer cells, thereby enhancing its therapeutic effects. miR-34a is also a regulator of RTKs by affecting *MET* gene expression and plays a role in gastric cancer proliferation and metastasis [35, 47, 48]. Furthermore, miR-34a regulates downstream signaling pathways such as PI3K/Akt and Wnt/β-catenin [49, 50].

miR-7 is a well-studied regulatory microRNA that has been extensively researched. Its target genes such as EGFR and its downstream effectors, protein kinase B (Akt) and extracellular kinase regulator ½ (ERK ½), are associated with RTK [51]. Studies on breast cancer cell lines have demonstrated that miR-7 plays a role in regulating various cellular processes, including EMT, metastasis, tumor-associated angiogenesis, as well as increased radiosensitivity [52–54]. In gastric cancer, miR-7 directly inhibits the expression of IGF1R, EGFR, and mTOR, which are the downstream effectors of the PI3K/Akt pathway. This leads to increased sensitivity to cisplatin treatment, apoptosis, as well as negative regulation of metastasis and proliferation in gastric cancer tissues [55–58]. The under-expression of miR-7 is considered a poor prognostic indicator and therapeutic delivery of this microRNA has been shown to reduce vasculogenesis and inflammation in gastric cancer [59]. miR-7 influences a variety of RTK-associated signaling pathways including EGFR downstream pathways Ras/Raf/MEK/ERK1/2 and PI3K/Akt/mTOR, as well as IGF1R/IRS pathway. It is also implicated in gefitinib-mediated cytotoxicity by affecting the expression of these molecular components [60, 61]. Additionally, miR-7 acts as a mediator in regulating cellular proliferation, migration, and invasion in non-small cell lung cancer (NSCLC) through the ERK/MAPK signaling pathway [62].

In addition to miR-7, other microRNAs such as miR-146a, miR-375, and miR-495, target the EGFR-encoding mRNAs [48, 63, 64]. miR-146a is a regulator of cellular proliferation, apoptosis, and invasion. Its expression is down-regulated in gastric cancer tissue samples compared to controls [65–68]. The expression levels of miR-146a show a 98% sensitivity in distinguishing affected tissue from normal surroundings. In addition, higher expression of miR-146a indicates a better response to chemotherapy [69]. miR-375 targets ErbB2 and EGFR subtype, along with the receptor d’Origine Natais (RON) RTK, and the downstream signaling effector JAK2. It is involved in regulating cellular proliferation, migration, and invasion in gastric cancer. Higher levels of miR-375 are also an indicator of favorable treatment response [64, 70, 71]. Moreover, lower serum levels of this microRNA serve as a marker of poor prognosis with an AUC of 0.871 and are associated with lymph node metastasis and a higher tumor stage [72]. miR-375 is under-expressed in NSCLC tissue and plasma samples, and is associated with lymph node and brain metastasis, advanced disease stage, and shorter overall survival [73–75]. Such a pattern of alteration has also been implicated in lung squamous cell carcinoma [76]. miR-495 is also another regulator of cellular apoptosis and migration through modulation of the Akt/mTOR pathway and ErbB2 expression [47, 48]. On the other hand, miR-206 mediates the regulation of c-MET, IGF1R, and MAPK3’s expression in gastric cancer. It induces apoptosis and inhibits resistance to cisplatin, metastasis, and proliferation [77–79].

miR-145 is a microRNA that targets the PI3K/AKT pathway, which is a downstream signaling pathway of various RTKs. By inhibiting the expression of AKT3, miR-145 can enhance the response to treatment [80] and regulate the proliferation and migration of breast cancer cells [81]. Additionally, miR-145 also regulates the expression levels of IGF-1R, another RTK. Results of both in vitro and in vivo investigations on adenoviral transfection of the miR-145 encoding gene in breast cancer cells have shown promising results in inhibiting tumor growth [82].

On the other hand, there are microRNAs such as miR-21, miR-10b, miR-373, and miR-155 that are oncogenic and contribute to breast cancer progression. miR-10b targets HOXD10, which itself plays a role in cellular migration and angiogenic switch in malignant breast cancer. It is also highly expressed in the tumor vasculature. Upregulation of miR-10b expression is associated with breast cancer, as well as glioblastoma, colorectal cancer, and pancreatic adenocarcinoma [83]. miR-21 is also overexpressed in breast cancer tissues and induces metastasis and migration by targeting a variety of effectors like smad7 and PTEN [84, 85]. Both tissue and serum levels of miR-21 have been extensively investigated as potential biomarkers for breast cancer, showing significant associations with a variety of clinicopathological characteristics including tumor grade, risk of metastasis, and hormonal receptor expression profiles [86]. miR-21 has also been extensively studied in hepatocellular carcinoma (HCC) and lung cancer. In HCC, miR-21 targets the tumor suppressor protein PTEN and its overexpression has been observed in serum samples [87, 88]. A meta-analysis by Qu et al. investigating the use of serum miR-21 as a diagnostic biomarker for HCC reported a pooled sensitivity of 85.2%, specificity of 79.2%, and an AUC of 0.89, suggesting its potential for early disease detection [89]. In lung cancer, miR-21 is considered an oncogenic microRNA and has been found to be overexpressed in NSCLC tissues [90]. A recently conducted meta-analysis has estimated the pooled sensitivity and specificity of miR-21 in lung cancer diagnosis to be 77% and 86%, respectively, with an AUC of 0.87. In addition, this biomarker possesses prognostic values with a hazard ratio (HR) of 1.49 for overall survival [91]. Additionally, miR-155, which also regulates the expression of PTEN, is overexpressed in NSCLC tissues and is a predictor of poor outcome [92, 93]. A meta-analysis on the diagnostic and prognostic value of miR-155 reported a pooled sensitivity of 0.82, specificity of 0.78, AUC of 0.87, and a hazard ratio (HR) of 1.26 for poor overall survival [94].

The miR-155, which targets the SOCS1 gene, has shown heterogeneous results in various studies regarding its role in regulating and promoting breast cancer progression [95, 96]. Some studies have reported conflicting findings. However, higher levels of miR-155 have been detected in the serum of breast cancer patients compared to controls, suggesting its potential as a diagnostic biomarker. The validity of miR-155 as a biomarker was supported by an AUC of 0.89 and its elevated levels were significantly associated with tumor grade, stage, and size [97]. These discrepancies in study outcomes may be attributed to differences in study population and conditions. Therefore, it is crucial to conduct studies with controlled conditions and larger sample sizes to obtain more conclusive results.

miR-155 and miR-99a have also been indicated as potential biomarkers for HCC diagnosis. miR-99a, a tumor suppressor, is downregulated in HCC and targets IGF-1R and mTOR. Both microRNAs show promise as diagnostic and prognostic biomarkers, with AUC of 0.799 and 0.84, respectively, according to the findings of a recently conducted meta-analysis [98, 99]. Additionally, high levels of these microRNAs are significantly associated with poor survival time. Furthermore, miR-99a has been explored as a therapeutic approach in both in vitro and in vivo studies, utilizing nano-particle delivery systems [100].

MicroRNAs play a crucial role in regulating various metastasis-associated cellular processes in colorectal cancer (CRC). Among these processes, the EMT is regulated by miR-612, which targets AKT2, and miR-200b, miR-200c, and miR-141, which target ZEB1 and ZEB2. In metastatic CRC tissues, miR-612 is found to be under-expressed, while the miR-141, miR-200b, and miR-200c show patterns of over-expression compared to control tissues [101, 102]. Additionally, miR-1249, miR-590-5p, miR-206, and miR-126 are involved in regulating angiogenesis and hypoxia response by inhibiting the expression of VEGFA. These microRNAs are relatively under-expressed in metastatic CRC tissues. Furthermore, miR-25-3p and miR-143 targets the RTKs VEGFR2 and IGF1R, respectively, to regulate the aforementioned cellular processes [103–108]. miR-18a and Let-7c target KRAS mRNA and regulate cellular proliferation and invasion, respectively [109, 110]. Another important regulator, miR-124, targets STAT3 and is found to be downregulated in CRC tissue samples, which is a predictor of poor prognosis. In a study by Wang et al., the hazard ratio of the downregulated levels of miR-124 for overall survival and disease-free survival were estimated at 4.634 (p = 0.002) and 4.533 (p = 0.002), respectively [111].

miR-126 plays a significant role in the progression of lung cancer by targeting various genetic components. The Reduction in STAT3 expression levels results in induction of cellular proliferation and migration, as well as decreased susceptibility to apoptosis, as indicated by the under-expression of caspase 3 in NSCLC [112]. Furthermore, miR-126-mediated inactivation of the VEGFA/VEGFR2/ERK pathway is associated with apoptosis and inhibition of metastatic characteristics in NSCLC [113]. Apart from these processes, EMT and angiogenesis are also modulated by miR-126 via targeting PI3K/AKT/Snail and VEGF/FGF-mediated signaling pathways, respectively [114, 115].

The down-regulation of miR-125a-3p is a key factor in determining poor prognosis in NSCLC and is significantly correlated with tumor size and metastasis. Conversely, higher expression of this marker is associated with higher overall and disease-free survival rates in NSLC patients [116]. Similar to miR-126, miR-125a inhibits cellular proliferation and metastasis in NSCLC by targeting STAT3 [117]. It is also a modulator of the HER2 receptor in small cell lung cancer (SCLC), and its under-expression is associated with the anti-tumor effects of cytotoxic drugs in HER2-positive SCLC [118].

miRNA-1 is a tumor suppressor that is highly conserved and targets multiple pathways, including members of the tyrosine kinase receptor family such as c-met and EGFR [119]. The down-regulation of miRNA-1 expression has been associated with a variety of malignancies, including ovarian cancer, colorectal cancer, squamous cell carcinomas from different origins, osteosarcoma, prostate cancer, gastric cancer, lung cancer, and rhabdomyosarcoma [120–129]. These malignancies exhibit an up-regulation of the aforementioned tyrosine kinase receptors, contributing to the importance of miRNA-1 in their development and progression.

The significance of these alterations and their role in the development and advancement of malignancies has prompted the conduction of studies exploring the potential use of these non-coding RNAs as biomarkers and treatment approaches, yielding promising results. In addition to the studies discussed in this section, Table 1 provides a comprehensive summary of the most extensively investigated miRNA-based biomarkers. Moreover, Table 2 highlights some of the therapeutic applications of miRNAs that have been investigated in these studies.

**Table 1:** Summary of the studies conducted on application of commonly investigated miRNAs as biomarkers in solid tumors

Malignancy	MicroRNA	Target	N. of cases	Pattern of alteration	Application	Site of measurement	Validity	Associated determinants of prognosis	References
Breast cancer	miR-21	PTEN	102	Up-regulation	Diagnostic, prognostic	Serum	AUC = 0.721	Visceral metastasis (p < 0.001)	[209]
	miR-195	Raf1	210	Down-regulation	Diagnostic, treatment response	Serum	AUC = 0.859	–	[210, 211]
	miR-155	SOCS1	103	Up-regulation	Diagnostic, treatment response	Serum	AUC = 0.801	–	[95, 212]
Colorectal cancer	miR-21	PTEN	200	Up-regulation	Diagnostic	Serum	AUC = 0.802	–	[213]
	miR-92a	PTEN	200	Up-regulation	Diagnostic, prognostic	Serum	AUC = 0.786	Poor survival (p = 0.03)	[213, 214]
	miR-200c	ZEB1/ZEB2	446	Up-regulation	Prognostic	Serum	–	Higher disease stage, lymph node metastasis (p = 0.0026), distant metastasis (0.0023), overall survival (p = 0.0064), tumor recurrence (HR = 4.51, p = 0.005)	[102, 215]
Gastric cancer	miR-182	FOXO1	47	Down-regulation	Diagnostic	Serum	AUC = 0.898	–	[216, 217]
	miR-21	PTEN	50	Up-regulation	Diagnostic	Serum, PBMC	AUC = 0.912 (serum), AUC = 0.898 (PBMC)	–	[218]
	miR-106b	ALEX1	90	Up-regulation	Diagnostic	Plasma	AUC = 0.7733	–	[219, 220]
Pancreatic cancer	miR-21	PTEN	49	Up-regulation	Diagnostic	Plasma	AUC = 0.62	–	[221, 222]
	miR-7	MAP3K1	8	Down-regulation	Prognostic	Plasma	–	Advanced tumor stage, poor survival	[223, 224]
	200c	ZEB1/ZEB2	84	Up-regulation	Prognostic	Tissue	–	Poor overall survival (p = 0.013)	[102, 225]
HCC	miR-122	IGF1R	50	Up-regulation	Diagnostic, prognostic	Serum	AUC = 0.954	Favorite prognosis (p < 0.001)	[226, 227]
	miR-21	PTEN	126	Up-regulation	Diagnostic	Plasma	AUC = 0.953	–	[221, 228]
NSCLC	miR-21	PTEN	152	Up-regulation	Diagnostic, prognostic	Serum	AUC = 0.81	Tumor size (p = 0.001), higher TNM stage (p = 0.004)	[221, 229]
	miR-126	VEGF	112	Up-regulation	Diagnostic	Serum	AUC = 0.793	–	[230, 231]
	let7c	IGF1R	120	Down-regulation	Diagnostic	Plasma	AUC = 0.714	–	[232, 233]

**Table 2:** Summary of the studies conducted on the therapeutic application of miRNAs in solid tumors.

ncRNA	Pathway	Malignancy	Type of study	Results	References
miR-122	ADAM17 and cyclin G1	HCC	In vivo	Inhibition of migration, invasion, tumorigenesis, angiogenesis, and metastasis	[133, 134]
miR-21	PTEN	HCC	In vitro and in vivo	Decreased tumor cell proliferation, migration, and invasion	[135]
miR-34a	P53, RAS, CDK4,BCL2, c-MET, and MYC	NSCLC	Animal study	Suppressed tumor growth and enhancement in the survival rate	[138, 139]
let-7b	RAS, BCL2, c-MET, and MYC	NSCLC	Animal study	Enhancement in the survival rate	[139]
miR-200c	PRDX2, GAPB/NRF2, and SESN1	Lung cancer	Animal study	Radiosensitivity augmentation of cancer cells	[140]
miR-34	P53, RAS, BCL2, c-MET, and MYC	Solid tumors	Clinical trial	Multiple immune-related severe adverse events	[141, 142]
miR-29b	DNMT3B, CDK6 and MCL1	NSCLC	Animal study	Decrease in tumor dimensions	[143]
miR-16	EGFR	NSCLC	Clinical trial	5% of the patients showed partial response	[144]
miR-125a-5p	HDAC4	Breast Cancer	Animal study	Decrease in tumor development, metastasis, and vasculature	[146]
miR-34a	NOTCH1 signaling pathway, Cyclin E2, and c-MYC	Breast Cancer	Animal study	Enhanced response to chemotherapy	[137, 147]

The limited effectiveness of current therapeutic interventions contributes to the unfavorable prognosis of HCC [130]. Synthetic miRNA antagonists or mimics, when administered intravenously, tend to accumulate in the liver and kidney. This characteristic renders liver cancer an appropriate model for evaluating the efficacy of miRNA-based therapeutic strategies [131, 132]. Biodistribution studies of nanoparticles indicate that a significant proportion of administered nanomaterials tend to accumulate in the liver prior to undergoing hepatic clearance, rendering it a favorable target for various delivery systems. In cases of liver cancer, miR-122, which is known for its high abundance and liver-specific expression, has been observed to be downregulated. Restoring miR-122 through the use of a lentiviral expression vector has been shown to inhibit invasion, tumorigenesis, and metastasis in vivo. The inhibition of HCC metastasis is achieved through the modulation of ADAM17 and cyclin G1 by miR-122 [133, 134]. On the other hand, the expression of miR-21 is significantly upregulated in HCC. Inhibition of miR-21 in cultured HCC cells leads to increased expression of the PTEN tumor suppressor and decreased tumor cell proliferation, migration, and invasion [135, 136].

Lung cancer is a prominent contributor to cancer-associated mortality worldwide, with 5-year survival rates ranging from 4 to 17%. Liposomes derived from lung surfactants offer a practical option for delivering drugs to the lungs, making them suitable vehicles for miRNA-targeting agents. The downregulation of miR-34a, a miRNA known for its tumor suppressor function, has been observed in different types of solid tumors, including lung cancer. A study conducted by Wiggins et al. demonstrated that the administration of synthetic miR-34a encapsulated in liposomes effectively suppressed tumor growth in mice with NSCLC. Importantly, this treatment approach exhibited no signs of immunogenicity or toxicity. These findings align with prior in vitro studies conducted on genetic variations of NSCLC cell lines, which demonstrated that miR-34a decreased cell proliferation and the formation of cell colonies through the p53 pathways [137, 138]. Kasinski et al. introduced a novel approach involving the utilization of NOV340 liposomes for the concurrent delivery of tumor suppressors miR-34 and let-7b in NSCLC. The implementation of this method led to a notable 40% improvement in the survival rate of mutant mice [139]. Furthermore, the utilization of miR-200c loaded-NOV340 liposomes has been shown to augment the radiosensitivity of lung cancer cells through the upregulation of oxidative stress response mechanisms and inhibiting DNA double-strand breaks caused by radiation [140].

In 2013, the initial phase of clinical testing involved the administration of MRX34, which consisted of miR-34 mimics enclosed within NOV340 liposomes. This therapeutic approach represented the first instance of utilizing miRNA for therapeutic purposes. Nevertheless, the study was terminated in 2016 as a result of the occurrence of significant unfavorable incidents among the participants [141, 142]. The research conducted by Wu et al. revealed that cationic lipoplexes based on DOTMA demonstrated a high level of efficacy in transporting miR-29b to both NSCLC cells and xenograft mouse models. Following a series of repeated administrations, the mice exhibited a notable decrease in tumor dimensions and a substantial increase in miR-29b expression, reaching a five-fold amplification specifically within the tumor tissue. This observation indicates that the release of miR-29b from DOTMA lipoplexes is highly effective [143]. In a phase I clinical trial, nonliving bacterial nano-cells were employed as vehicles for the administration of miR-16 to patients with NSCLC. The system specifically targeted cancer cells expressing EGFR, resulting in inhibited tumor growth. Nevertheless, there were documented toxicities that were dependent on the dosage, such as anaphylaxis, inflammation, and cardiac events. The study recorded varying response rates, with 5% of participants exhibiting partial response, 68% experiencing stable disease, and 27% demonstrating progressive disease [144].

Given that HER-2 positive breast cancers constitute approximately 30% of cases characterized by an unfavorable prognosis, there is an increasing focus on effectively targeting this overexpressed receptor. Within this particular framework, experiments conducted on live mice with breast cancer have shown that the introduction of the tumor suppressor miR-125a-5p through lentiviral delivery resulted in a decrease in tumor development, metastasis, and vasculature. This effect was achieved by specifically targeting histone deacetylase 4 (HDAC4). Hayward et al. demonstrated that introducing miR-125a-5p into hyaluronic acid (HA)-coated liposomes effectively suppressed the HER-2 proto-oncogene in 21MT-1 breast cancer cells through transfection. Consequently, the inactivation of the MAPK and PI3K/AKT signaling pathways led to decreased migratory and proliferative capabilities [145, 146]. The utilization of HA/miRNA nanoparticles has shown potential in the context of targeted clinical interventions for breast cancer. The study by Deng et al. employed HA-chitosan nanoparticles for the simultaneous encapsulation of doxorubicin and miR-34a, resulting in an improved response to chemotherapy and a reduction in cancer cells migration [147, 148]. The in vivo experiments demonstrated a significant 58% decrease in tumor volume upon the incorporation of miR-9, miR-21, and miR-145 sponges into magnetic particles within PEI particles [149, 150]. The study conducted by Panebianco et al. discovered that the utilization of silica nanoparticles facilitated the delivery of miR-34a into mammary tumors, resulting in reduced tumor growth in mice. The diminished expression of target genes, such as c-Myc, served as an indicator of the biological efficacy of the administered miR-34a [137].

## tsRNAs and piRNAs role in RTK pathway in cancer

Transfer RNA-derived small RNAs (tsRNAs) are derived from tRNAs. The biogenesis of tsRNAs and their downstream mechanisms of action are still being studied, but it has been observed that they can be involved in regulating gene expression and translation, similar to miRNAs. tsRNAs have been found to be associated with Argonaute proteins and can mediate translational repression of mRNAs via binding to target 3' UTRs. Interestingly, a recent study has shown that a tsRNA derived from a leucine tRNA plays a role in global protein translation by regulating the expression of genes coding for ribosomal components [151]. Some tsRNAs have been found to be oncogenic, while others have been found to be tumor-suppressive. Therefore, tsRNAs have the potential to be a new target for anticancer therapy.

Emerging evidence suggests that piRNAs are involved in RTK signaling in solid tumors. piRNAs, a class of small ncRNA molecules typically 21–35 nucleotides in length, are primarily associated with the PIWI subfamily of Argonaute proteins and are involved in transposon silencing during germline development. In mammals, piRNAs are generated from long, single-stranded transcripts that are clustered throughout the genome, with approximately 20,000 piRNAs present in the human genome. While piRNAs were initially thought to function only in gonadal cells, recent research has shown that they are also expressed in somatic tissues, although at low levels, and misexpressed in cancers. Although the precise functional roles of piRNAs in RTK signaling in solid tumors are not fully understood, recent studies have suggested that these small ncRNAs may have therapeutic potential as targets in cancer treatment and may serve as useful biomarkers for diagnosis and prognosis [152, 153].

## Long non-coding RTK-RNAs

### Linear lncRNAs role in RTK pathway in cancer

**Fig. 3:**
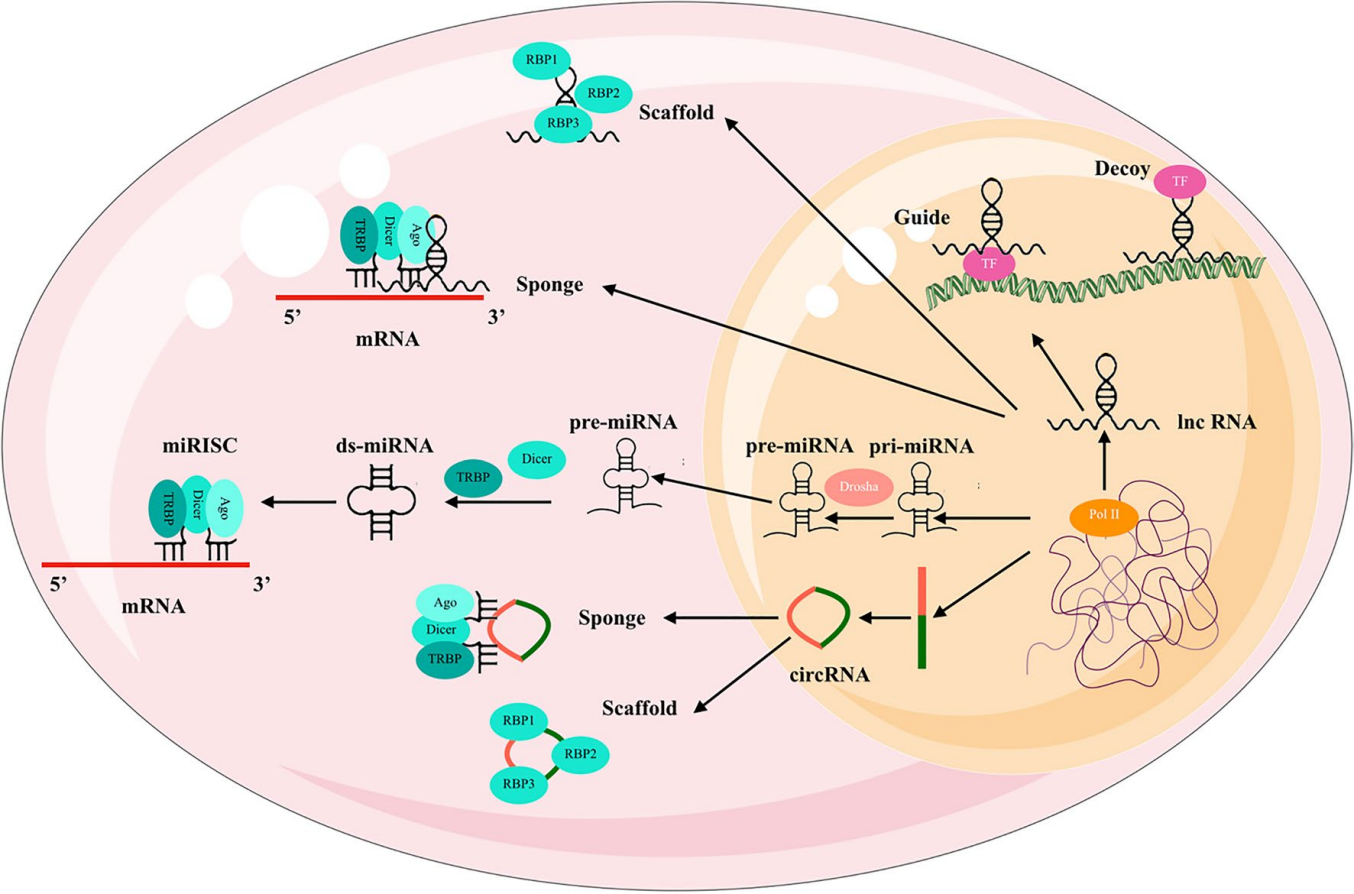
Biogenesis and mechanism of action in Non-coding RNAs (TF = Transcription factor). This figure was created using the Servier Medical Art Commons Attribution 3.0 Unported License (http://smart.servier.com, accessed on 11 July, 2023)

lncRNAs are a class of regulatory molecules that are longer than 200 nucleotides with a fundamental role in gene expression through their interaction with chromatin, transcriptional machinery, and other cellular components. Their biogenesis begins with transcription, predominantly by RNA polymerase II, similar to messenger RNAs (mRNAs). The initial transcripts, known as primary lncRNAs (pri-lncRNAs), often undergo splicing, polyadenylation, and capping [154]. However, lncRNA transcripts can exhibit unique features, such as variable splicing and less efficient polyadenylation, which can impact their stability and cellular localization [155]. Following successful processing, mature lncRNAs participate in various cellular processes. For instance, nuclear lncRNAs can interact with chromatin and transcription factors, thereby affecting transcriptional regulation [156]. Cytoplasmic lncRNAs, on the other hand, can modulate mRNA stability or translation, interact with miRNAs, or influence signal transduction pathways [157] (Fig. 3).

Regarding the interaction between lncRNA and microRNAs in promoting RTK expression, extensive research has been conducted in various types of cancer, such as colon, gastric, breast, osteosarcoma, HCC, and NSCLC [158–161]. For instance, lncRNA XIST has been found to upregulate RTK family member, AXL, via sponging miR-93-5p, resulting in the overexpression of hypoxia-inducible factor 1-alpha (HIF1A). Ultimately, this process participated in enhancing the tumorigenesis capacity in colon cancer cells [162]. On the other hand, lncRNA XIST expression can also be involved in the upregulation of RTK-like orphan receptor 1 (ROR1) by sponging miR-30a-5p, leading to promoting cellular growth [163]. Another study demonstrated that ERBB4, a member of the RTK family, is modulated by lncRNA, called Linc00152. Mechanistically, lncRNA Linc00152 regulates the expression of ERBB4 by targeting miR-193a-3p, which may hinder the sensitization of malignant cells toward oxaliplatin-based chemotherapy. Targeted downregulation of Linc00152 expression significantly reduces chemoresistance in malignant cells, suggesting promising implications for developing novel therapeutic strategies [164]. Another lncRNA, known as H19, acts on the ubiquitin ligase E3 family by modulating miR-675 to promote RTK stability, thereby contributing to breast cancer development [165].

Furthermore, in neuroblastoma, lncRNA MALAT1 has been discovered to promote cellular invasion and migration via upregulating AXL, an RTK member. Inhibition of AXL has emerged as a promising therapeutic approach [166]. Similarly, the upregulation of a novel lncRNA called CALIC has been identified to enhance colon cancer cell migration and metastasis through regulating AXL signaling [167]. Moreover, another investigation found that in anaplastic thyroid cancer, lncRNA MALAT1 overexpression can lead to the activation of the RTK signaling pathway via a cascade of intermediary cellular signaling pathways, offering a remarkable diagnostic advantage [168]. In breast cancer, lncRNA MAYA has been found to play a role in bone metastasis. Regarding the underlying mechanism, it has been demonstrated that lncRNA MAYA can stimulate the formation of a heterodimeric complex consisting of ROR1, HER3, and the other RTKs, which subsequently activate signaling cascades and contribute to cancer cell migration [169]. Another lncRNA, SPRY4-IT1, has been shown to regulate the expression of the RTK family member FGFR2 by interacting with chromatin and regulating the expression of nearby genes. Additionally, SPRY4-IT1 has a regulatory effect on EMT, which is considered an essential mechanism in cell migration [170].

In addition to lncRNAs, which act as activators of the RTK signaling pathway, there are several lncRNAs with inhibitory effects on RTK regulatory pathways, resulting in the suppression of tumor development and progression. For example, a lncRNA named LINC00526 could function as a tumor suppressor that establishes a negative feedback loop with AXL, indicating its potential therapeutic application in glioma treatment [171]. Conversely, RTK can interfere with the functionality of lncRNAs, thereby impacting cancer metastasis. In breast cancer, for example, EGF has been found to decrease the expression of a newly discovered lncRNA called LIMT, which subsequently promotes tumor invasion [172]. Tumor-suppressing lncRNAs also restrict the invasive potential of melanoma and HCC by inhibiting RTK-related pathways [173]. Interestingly, Eph tyrosine kinase receptor A4 functions as a suppressor of the EMT process, in which lncRNA TUSC acts as a sponge to sequester miR-10a and increase Eph expression, thereby restricting tumor growth. This pathway exhibits similarities to previous studies but with the opposite effect [174].

On the other hand, it has been demonstrated that RTK inhibitors (RTKIs) are effective therapeutic agents for treating cancer. In this context, researchers have identified 45 lncRNAs that are differentially expressed in NSCLC cells, which are resistant to epidermal growth factor receptor tyrosine kinase inhibitors (EGFR-TKIs). The downregulated LINC01128, acting as a specific miRNA sponge, decreases PTEN via sponging miR-25-3p. This activates the PI3K/Akt signaling pathway and promotes EGFR-TKI resistance. Another lncRNA, HIF1A-AS2, has been shown to be downregulated by RTKIs, resulting in reduced tumor growth in NSCLC [175]. HIF1A-AS2 also regulates the expression of specific genes involved in angiogenesis, such as VEGFA, by interacting with specific proteins. By inhibiting HIF1A-AS2 expression, RTKIs can also indirectly decrease the expression of these angiogenic factors, further inhibiting tumor growth [176]. Similarly, a study investigated the potential of a lncRNA called growth arrest-specific 5 (GAS5) in improving the efficacy of EGFR-TKIs in NSCLC therapy. The results showed that GAS5 is downregulated in lung adenocarcinoma tissues, and lower expression levels are associated with larger tumor sizes, poor differentiation, and advanced pathological stages. Furthermore, overexpression of GAS5 sensitizes resistant NSCLC cells to EGFR-TKIs and inhibits tumor growth in mice treated with gefitinib. These findings suggest that GAS5 may be a potential biomarker for diagnosing lung adenocarcinoma and a possible therapeutic target to reverse EGFR-TKI resistance [177]. Moreover, the lncRNA H19 has been shown to be involved in the regulation of the EGFR signaling pathway. H19 regulates EGFR signaling by binding to the EGFR protein and promoting its degradation. Therefore, lncRNA H19 may play a key role in the response of cancer cells, such as NSCLC, to EGFR-TKIs. Thereby, lncRNA H19 seems to be a key factor in regulating RTK signaling [178]. RTKIs with a similar effect on the mentioned pathways on UCA1 [179], CRNDE [180], and PCAT1 [181] genes lead to the development of other types of NSCLCs. In an alternative mechanism, LncRNA-SARCC functions as a suppressor of the androgen receptor (AR) protein, hindering the propagation of its downstream signals involving AKT, MMP-13, K-RAS, and P-ERK. This obstruction leads to the inhibition of invasiveness in RCC cells while also enhancing their sensitivity to Sunitinib therapy. The findings of this investigation underscore the potential effectiveness of targeting LncRNA-SARCC and its underlying pathway as a viable therapeutic approach for treating RCC. It should be noted, however, that certain RTKIs have been shown to induce the expression of a group of lncRNA molecules, including LncRNA-SARCC, which are associated with improved prognosis and contribute to the restoration of cancer suppression in RCC [182]. Therefore, this evidence could imply that lncRNAs may play an essential role in regulating RTKs.

Altogether, lncRNAs play a crucial role in modulating RTK signaling pathways and the development of cancer. However, comprehensive investigations are necessary to elucidate the intricate mechanisms underlying lncRNA-mediated regulation of RTK signaling and to recognize potential therapeutic targets for improving RTK-associated diseases. Nonetheless, gaining a deeper understanding of these pathways offers a new approach to managing different types of cancer, highlighting the need for more comprehensive investigation (Tables 3, 4).

**Table 3:** Summary of the studies conducted on the application of commonly investigated lncRNAs as biomarkers in solid tumors

Malignancy	lncRNA	Target	N. of cases	Pattern of alteration	Application	Site of measurement	Validity	Associated determinants of prognosis	Further information	References
NSCLC	TMPO-AS1	ERBB2	30	Up-regulation	Prognostic	Serum	_	_	LncRNA TMPO-AS1 facilitates the proliferation and metastasis of NSCLC cells by up-regulating ERBB2 via sponging miR-204-3p	[160]
NSCLC	DANCR	ERBB2	84	Up-regulation	Prognostic	Serum	AUC = 0.8966	Shorter survival rate (p < 0.0001), lymph node metastasis (p = 0.001)	lncRNA DANCR promoted non-small cell lung cancer cells metastasis via modulating of miR-1225-3p/ErbB2 signal	[234]
Gastric cancer	MIR137HG	MET	69	Up-regulation	Diagnostic	Tissue	AUC = 0.667	Lymph node metastasis p = 0.063	miR-2682-3p antagonizes its host lncRNA-MIR137HG by interacting with the same target FUS to regulate the progression of gastric cancer	[235]
Colon cancer	CALIC	AXL		Up-regulation	_	Tissue	_	_	The novel lncRNA CALIC upregulates AXL to promote colon cancer metastasis	[167]
Colon cancer	XIST	AXL	36	Up-regulation	Diagnostic and therapeutic	Serum	_	_	LncRNA XIST modulates HIF‐1A/AXL signaling pathway by inhibiting miR‐93‐5p in colorectal cancer	[162]
Colon cancer	XIST	ROR1	294	Up-regulation	Therapeutic	Serum		Lymphatic metastasis: P = 0.015, poorer prognosis: P = 0.003	Atractylenolide II reverses the influence of lncRNA XIST/miR‐30a‐5p/ROR1 axis on chemo‐resistance of colorectal cancer cells	[163]
Neuroblastoma	MALAT1	AXL	62	Up-regulation	Prognostic	Serum	_	Enhancement of the cellular invasion: p < 0.01	LncRNA-MALAT1-mediated Axl promotes cell invasion and migration in human neuroblastoma	[166]
Melanoma	TINCR	AXL	95	Down-regulation	_	Serum	_	_	lncRNA TINCR attenuates the proliferation and invasion, and enhances the apoptosis of cutaneous malignant melanoma cells by regulating the miR-424-5p/LATS1 axis	[236]
Melanoma	GAS6-AS2	AXL	85	Up-regulation	Prognostic	Serum	_	Prognosis: p < .0001	Increased expression of long noncoding RNA GAS6-AS2 promotes proliferation and inhibits apoptosis of melanoma cells via upregulating GAS6 expression	[237]
Osteosarcoma	LINC00852	AXL	34	Up-regulation	Prognostic	Serum	_	Survival rates: P = .000	Exosome-transmitted linc00852 associated with receptor tyrosine kinase AXL dysregulates the proliferation and invasion of osteosarcoma	[159]
HCC	HULC	MET	42	Up-regulation	Diagnostic and therapeutic	Serum	_	_	Long noncoding RNA HULC promotes hepatocellular carcinoma progression	[161]
HCC	TUSC	Eph	75	Down-regulation	Prognostic	Serum [171]	_	3-year overall survival and disease-free survival in HCC patients: *p* = 0.007 and 0.015 respectively	lncRNA TUSC7 acts a molecular sponge for miR-10a and suppresses EMT in hepatocellular carcinoma	[174]
Glioma	LINC00526	AXL	52	Down-regulation	Prognostic and therapeutic	Serum	_	_	lncRNA LINC00526 represses glioma progression via forming a double negative feedback loop with AXL	[171]
Breast cancer	LIMT	EGF	44	Down-regulation	Prognostic	Serum	_	_	LIMT is a novel metastasis inhibiting lncRNA suppressed by EGF and downregulated in aggressive breast cancer	[172]
Breast cancer	H19	EGFR and c-met	500 (cells)	Up-regulation	Prognostic	Serum	_	_	H19 ncRNA-derived miR-675 enhances tumorigenesis and metastasis of breast cancer cells by downregulating c-Cbl and Cbl-b	[165]
Breast cancer	MAYA	HER3, ROR1	_	Up-regulation	Therapeutic	Serum	_	_	A ROR1-HER3-LncRNA signaling axis modulates the Hippo-YAP pathway to regulate bone metastasis	[169]
Thyroid cancer	MALAT1	RTKs	_	Up-regulation	Therapeutic	Serum	_	_	Transcript-level regulation of MALAT1-mediated cell cycle and apoptosis genes using dual MEK/Aurora kinase inhibitor “BI-847325” on anaplastic thyroid carcinoma	[168]
RCC	HOTAIR	AXL	86	Up-regulation	Diagnostic and therapeutic	Serum	_	_	LncRNA HOTAIR regulates HIF-1*α*/AXL signaling through inhibition of miR-217 in renal cell carcinoma	[158]

**Table 4:** Summary of the studies conducted on the therapeutic application of lncRNAs in solid tumors

ncRNA	Target	Malignancy	Type of study	Results	References
TMPO-AS1	ERBB2	NSCLC	In vitro	LncRNA TMPO-AS1 facilitates the proliferation and metastasis of NSCLC cells by up-regulating ERBB2 via sponging miR-204-3p	[160]
DANCR	ERBB2	NSCLC	In vitro	LncRNA DANCR promoted non-small cell lung cancer cells metastasis via modulating of miR-1225-3p/ErbB2 signal	[234]
MIR137HG	MET	Gastric cancer	In vitro	MiR-2682-3p antagonizes its host lncRNA-MIR137HG by interacting with the same target FUS to regulate the progression of gastric cancer	[235]
CALIC	AXL	Colon cancer	In vivo	The novel lncRNA CALIC upregulates AXL to promote colon cancer metastasis	[167]
XIST	AXL	Colon cancer	In vivo*/*in vitro	LncRNA XIST modulates HIF‐1A/AXL signaling pathway by inhibiting miR‐93‐5p in colorectal cancer	[162]
XIST	ROR1	Colon cancer	Clinical trial	Atractylenolide II reverses the influence of lncRNA XIST/miR‐30a‐5p/ROR1 axis on chemo‐resistance of colorectal cancer cells	[163]
MALAT1	AXL	Neuroblastoma	In vitro	LncRNA-MALAT1-mediated Axl promotes cell invasion and migration in human neuroblastoma	[166]
TINCR	AXL	Melanoma	In vivo*/*in vitro	LncRNA TINCR attenuates the proliferation and invasion, and enhances the apoptosis of cutaneous malignant melanoma cells by regulating the miR-424-5p/LATS1 axis	[236]
GAS6-AS2	AXL	Melanoma	In vivo*/*in vitro	Increased expression of long noncoding RNA GAS6-AS2 promotes proliferation and inhibits apoptosis of melanoma cells via upregulating GAS6 expression	[237]
LINC00852	AXL	Osteosarcoma	In vivo*/*in vitro	Exosome-transmitted linc00852 associated with receptor tyrosine kinase AXL dysregulates the proliferation and invasion of osteosarcoma	[159]
HULC	MET	HCC	In vivo*/*in vitro	The lncRNA “highly upregulated in liver cancer” (HULC) promotes MET expression through sponging miR-2052 in HCC	[161]
TUSC	Eph	HCC	In vitro	LncRNA TUSC7 acts a molecular sponge for miR-10a and suppresses EMT in hepatocellular carcinoma	[174]
LINC00526	AXL	Glioma	In vitro	LncRNA LINC00526 represses glioma progression via forming a double negative feedback loop with AXL	[171]
LIMT	EGF	Breast cancer	In vivo*/*in vitro	LIMT is a novel metastasis inhibiting lncRNA suppressed by EGF and downregulated in aggressive breast cancer	[172]
H19	EGFR and c-met	Breast cancer	In vitro	H19 ncRNA-derived miR-675 enhances tumorigenesis and metastasis of breast cancer cells by downregulating c-Cbl and Cbl-b	[165]
MAYA	HER3, ROR1	Breast cancer	In vivo*/*in vitro	The orphan receptor tyrosine kinase ROR1 can form heterodimers with other RTKs, such as HER3, to activate signaling pathways that promote cancer cell proliferation, survival, and invasion	[169]
MALAT1	RTKs	Thyroid cancer	In vitro	Transcript-level regulation of MALAT1-mediated cell cycle and apoptosis genes using dual MEK/Aurora kinase inhibitor “BI-847325” on anaplastic thyroid carcinoma	[168]
HOTAIR	AXL	RCC	In vivo*/*in vitro	LncRNA HOTAIR regulates HIF-1*α*/AXL signaling through inhibition of miR-217 in renal cell carcinoma	[158]

## Circular lncRNAs role in RTK pathway in cancer

**Fig. 4:**
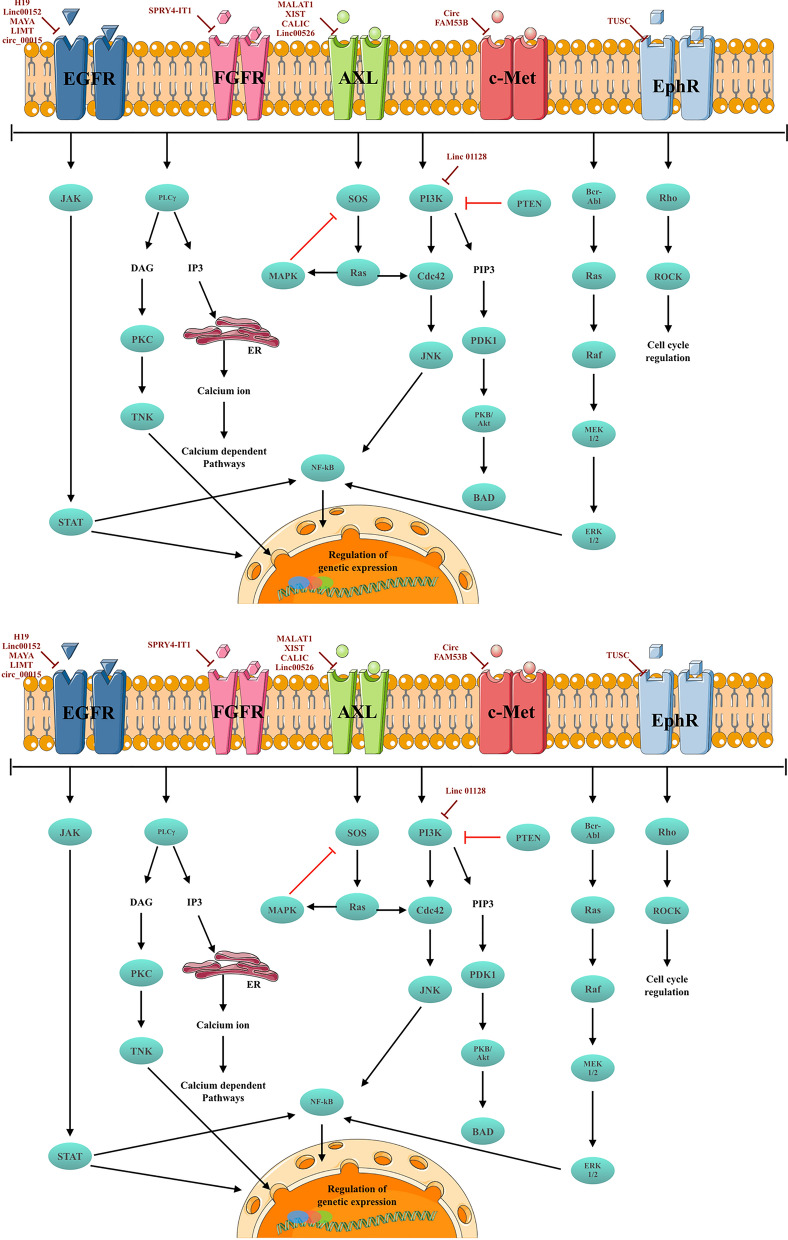
RTK-mediated signaling pathways and associated lncRNAs and circRNAs in solid tumors. lncRNAs and circRNAs target various components of these pathways, including both receptors and downstream signaling cascades. This figure was created using the Servier Medical Art Commons Attribution 3.0 Unported License (http://smart.servier.com (accessed 10 July 2023))

CircRNAs are a novel class of endogenous lncRNAs that form covalently closed continuous loops and are generated from pre-mRNA back-splicing events, where a downstream splice donor is joined with an upstream splice acceptor [183]. The biogenesis of circRNAs is facilitated by several factors, including the presence of complementary Alu sequences and RNA-binding proteins like Quaking and Muscleblind [184]. The regulation of circRNA biogenesis, although not fully elucidated, is believed to be influenced by both cis-regulatory elements and trans-acting factors [185]. Importantly, circRNAs have been implicated in various biological processes, including acting as microRNA sponges and interacting with RNA-binding proteins [186] (Fig. 4).

Several studies have investigated the association between circular RNA and receptor tyrosine kinase in lung adenocarcinoma. One study demonstrated that hsa_circ_0070661 suppressed cancer progression by regulating the miR-556-5p/TEK axis [187]. Li et al. found that circ_0001058 inhibited lung adenocarcinoma development by modulating the miR-486-5p/TEK signaling pathway [188]. Moreover, Wang et al. discovered that overexpression of hsa_circ_0012673 promoted tumor proliferation by acting as a sponge for miR-22, which led to the upregulation of TEK expression [189]. Overall, these findings suggest that circRNAs regulate TEK signaling in lung adenocarcinoma and may serve as potential therapeutic targets for this disease. Studies have demonstrated a correlation between circular RNA and receptor tyrosine kinase in promoting glioma progression. For instance, circFAM53B promotes glioma proliferation and metastasis by activating the c-MET/PI3K/AKT pathway via sponging miR-532-3p [190]. Circ_0001588 upregulates ERBB4 to promote glioma malignant progression through sponging miR-128, [191] while circ_0001162 enhances cell proliferation and invasion of glioma through the miR-936/ERBB4 axis [192]. Furthermore, circRNA EPHB4 modulates stem properties and proliferation of gliomas via sponging miR-637 and upregulating SOX10, indicating that the RTK pathway may be involved in glioma development and progression mediated by circRNA-miRNA interactions [193]. These results highlight the potential of circRNA as a therapeutic target for glioma treatment and provide insights into the underlying mechanisms of glioma pathogenesis. Also, in other research, various types of cancer have been explored. For example, Circ_0044520 has been found to regulate the progression of laryngeal squamous cell carcinoma via the miR-338-3p/ROR2 axis [194]. Another study shows that circRNA_0006470 promotes gastric cancer cell proliferation and migration by functioning as a sponge of miR-27b-3p [195]. Additionally, tumor-derived exosomal circRNA_102481 has been shown to contribute to EGFR-TKIs resistance in non-small cell lung cancer via the miR-30a-5p/ROR1 axis [196]. NSD2 circular RNA has been found to promote colorectal cancer metastasis by targeting miR-199b-5p-mediated DDR1 and JAG1 signaling [197]. The study by Zheng et al. found that Circ_0079558 promotes papillary thyroid cancer progression by binding to miR-26b-5p, activating MET/AKT signaling [198]. Wang et al. found that the RNA-binding protein IGF2BP2 enhances the plenty of circ_0000745 and promotes the aggressiveness and stemness of ovarian cancer cells by activating the microRNA-3187-3p/ERBB4/PI3K/AKT signaling pathway [199]. Furthermore, circ_LAMP1 has been found to promote T-cell lymphoblastic lymphoma progression by acting as a competing endogenous RNA (ceRNA) for miR-615-5p to regulate DDR2 expression (200).

In developing resistance to specific cancer therapies, circular RNA correlates with receptor tyrosine kinase, as recent studies indicate. A study conducted on lung adenocarcinoma cells found that hsa_circ_0007312 promotes third-generation EGFR-TKI resistance through pyroptosis and apoptosis via the miR-764/MAPK1 axis [201]. Another study identified circ_0014235 as a factor responsible for conferring Gefitinib resistance and malignant behaviors in non-small cell lung cancer (NSCLC) by governing the miR-146b-5p/YAP/PD-L1 pathway [202]. Similarly, hsa_circ_0005576 promotes osimertinib resistance through the miR-512-5p/IGF1R axis in lung adenocarcinoma cells [203]. In contrast, Propofol was shown to suppress lung cancer tumorigenesis by modulating the circ-ERBB2/miR-7-5p/FOXM1 axis [204]. According to a study on non-small cell lung cancer patients receiving Gefitinib therapy, the circular RNA hsa_circ_0109320 showed significantly elevated expression levels in individuals who positively responded to EGFR-TKI treatment [205]. The study by Wang et al. revealed a novel protein encoded by circASK1 that plays a crucial role in overcoming gefitinib resistance in lung adenocarcinoma by competitively activating apoptosis signal-regulating kinase 1 (ASK1)-dependent apoptosis [206]. Finally, circRNA_001895 was found to promote sunitinib resistance to renal cell carcinoma through the regulation of apoptosis and DNA damage repair [207]. These findings have shown that circRNAs can play a crucial role in developing drug resistance in cancer cells, and further research is needed to explore their therapeutic potential (Tables 5, 6).

**Table 5:** Summary of the studies conducted on the application of commonly investigated circRNAs as biomarkers in solid tumors

Malignancy	CircRNA	Target	N. of cases	Pattern of alteration	Application	Site of measurement	Validity	Associated determinants of prognosis	Additional information	References
Lung adenocarcinoma	Hsa_circ_0070661	TEK	38	Up-regulation	Prognostic	Serum	–	–	Hsa_circ_0070661 inhibits cancer progression through miR-556-5p/TEK axis in lung adenocarcinoma	[187]
	Circ_0001058	TEK		Up-regulation	Prognostic	Serum	–	–	Circ_0001058 represses the progression of lung adenocarcinoma through governing of the miR-486-5p/TEK signaling axis	[188]
	circ7312	EGFR-TKI	89	Up-regulation	Prognostic, treatment response	Tumor tissue	–	DFS and OS were significantly longer in LUAD patients with low circ7312 than in those with high circ7312 expression(p = 0.0043 and p = 0.0099)	Hsa_circ_0007312 Promotes Third-Generation Epidermal Growth Factor Receptor-Tyrosine Kinase Inhibitor Resistance through Pyroptosis and Apoptosis via the MiR-764/MAPK1 Axis in Lung Adenocarcinoma Cells. J Cancer	[201]
	circ-0003748	EGFR-TKI	23	Up-regulation	Prognostic	Plasma	–	–	Construction of a circRNA-miRNA-mRNA Regulated Pathway Involved in EGFR-TKI Lung Adenocarcinoma Resistance	[238]
	circ-0001398	EGFR-TKI	23	Up-regulation	Prognostic	Plasma	–	–	Construction of a circRNA-miRNA-mRNA Regulated Pathway Involved in EGFR-TKI Lung Adenocarcinoma Resistance	[238]
	hsa_circ_0005576	IGF1R	20 (mice)	Up-regulation	Prognostic, treatment response	Tumor tissue	–	–	Hsa_circ_0005576 promotes osimertinib resistance through the miR-512-5p/IGF1R axis in lung adenocarcinoma cells	[203]
	hsa_circ_0012673	ERBB3	33	Up-regulation	Prognostic		–	–	Increased circular RNA hsa_circ_0012673 acts as a sponge of miR-22 to promote lung adenocarcinoma proliferation	[189]
NSCLC	Circ_0014235	EGFR-TKI	24 (mice)	Up-regulation	Treatment response	Tumor cells	–	–	Circ_0014235 confers Gefitinib resistance and malignant behaviors in non-small cell lung cancer resistant to Gefitinib by governing the miR-146b-5p/YAP/PD-L1 pathway	[202]
	circRNA_102481	ROR1	58	Up-regulation	Diagnostic, treatment response	Serum	–	High expression of circRNA_1024810 in exosomes was closely associated with brain metastasis (P < 0.05)	Tumor-derived exosomal circRNA_102481 contributes to EGFR-TKIs resistance via the miR-30a-5p/ROR1 axis in non-small cell lung cancer	[196]
	hsa_circ_0109320	EGFR-TKI	52	Up-regulation	Diagnostic, treatment response	Plasma	AUC = 0.81	Elevated hsa_circ_0109320 expression was associated with significantly better PFS in patients with NSCLC after gefitinib treatment(p = 0.02)	Circular RNA profiling identified as a biomarker for predicting the efficacy of Gefitinib therapy for non-small cell lung cancer	[205]
Lung cancer	circ-ERBB2	ERBB2	31	Up-regulation	Diagnostic, treatment response	Cancer tissue	–	–	Propofol suppresses lung cancer tumorigenesis by modulating the circ-ERBB2/miR-7-5p/FOXM1 axis	[204]
Glioma	circFAM53B	c-met	40	Up-regulation	Treatment response	Glioma cells	–	Higher circFAM53B levels were linked to poorer survival of glioma patients (p = 0.0441)	CircFAM53B promotes the proliferation and metastasis of glioma through activating the c-MET/PI3K/AKT pathway via sponging miR-532-3p	[190]
	Circ_0001588	EPHB4		Up-regulation	Prognostic		–	–	. Circ_0001588 Upregulates ERBB4 to Promote Glioma Malignant Progression Through Sponging miR-1281	[191]
	circ_0001162	EPHB4	30	Up-regulation	Prognostic	Glioma cells	–	–	circ_0001162 promotes cell proliferation and invasion of glioma via the miR-936/ERBB4 axis	[192]
	hsa_circ_0081519	EPHB4	40	Up-regulation	Prognostic, treatment response	Glioma cells	–	Higher circEPHB4 or SOX10 level was independently correlated with reduced overall survival(p < 0.05)	. CircRNA EPHB4 modulates stem properties and proliferation of gliomas via sponging miR-637 and up-regulating SOX10	[193]
Laryngeal squamous cell carcinoma	Circ_0044520	ROR2	60	Up-regulation	Prognostic, treatment response	–	-	circ_0044520 was correlated with tumor size, clinical stage, and lymphoid node metastasis(p < 0.05)	. Circ_0044520 regulates the progression of laryngeal squamous cell carcinoma via the miR-338-3p/ROR2 axis	[194]
Ovarian cancer	circ_0000745	ERBB4	50	Up-regulation	Prognostic, treatment response	Tumor tissue	–	–	. RNA-binding protein IGF2BP2 enhances circ_0000745 abundancy and promotes aggressiveness and stemness of ovarian cancer cells	[199]
Gastric cancer	hsa_circ_0006470	ROR1		Up-regulation	Diagnosis, treatment response	Cancer cells	–	–	circRNA_0006470 promotes the proliferation and migration of gastric cancer cells by functioning as a sponge of miR-27b-3p	[195]
PTC	circ_0079558	MET	30	Up-regulation	Diagnostic	Tumor tissues	–	–	Circ_0079558 promotes papillary thyroid cancer progression by binding to miR-26b-5p to activate MET/AKT signaling	[198]
Colorectal cancer	circ-NSD2	DDR1		Up-regulation	Prognostic, treatment response		–	–	NSD2 circular RNA promotes metastasis of colorectal cancer by targeting miR-199b-5p-mediated DDR1 and JAG1 signalling	[197]
T-cell lymphoblastic lymphoma	circ-LAMP1	DDR2	34	Up-regulation	Prognostic	T-LBL tissue	–	–	Circ-LAMP1 promotes T-cell lymphoblastic lymphoma progression via acting as a ceRNA for miR-615-5p to regulate DDR2 expression	[200]

**Table 6:** Summary of the studies conducted on the therapeutic application of circRNAs in solid tumors

circRNA	Target	Malignancy	Type of study	Results	References
Hsa_circ_0070661	TEK + BB3:B23	Lung adenocarcinoma	In vivo*/*in vitro	Hsa_circ_0070661 inhibits cancer progression through miR-556-5p/TEK axis in lung adenocarcinoma	[187]
Circ_0001058	TEK	Lung adenocarcinoma	In vivo*/*in vitro	Circ_0001058 represses the progression of lung adenocarcinoma through governing of the miR-486-5p/TEK signaling axis	[188]
circ7312	EGFR-TKI	Lung adenocarcinoma	In vivo/Invitro	Hsa_circ_0007312 Promotes Third-Generation Epidermal Growth Factor Receptor-Tyrosine Kinase Inhibitor Resistance through Pyroptosis and Apoptosis via the MiR-764/MAPK1 Axis in Lung Adenocarcinoma Cells. J Cancer	[201]
circ-0003748	EGFR-TKI	Lung adenocarcinoma	In vitro	Construction of a circRNA-miRNA-mRNA Regulated Pathway Involved in EGFR-TKI Lung Adenocarcinoma Resistance	[238]
circ-0001398	EGFR-TKI	Lung adenocarcinoma	In vitro	Construction of a circRNA-miRNA-mRNA Regulated Pathway Involved in EGFR-TKI Lung Adenocarcinoma Resistance	[238]
hsa_circ_0005576	IGF1R	Lung adenocarcinoma	In vivo*/*in vitro	Hsa_circ_0005576 promotes osimertinib resistance through the miR-512-5p/IGF1R axis in lung adenocarcinoma cells	[203]
hsa_circ_0012673	ERBB3	Lung adenocarcinoma	In vitro	Increased circular RNA hsa_circ_0012673 acts as a sponge of miR-22 to promote lung adenocarcinoma proliferation	[189]
Circ_0014235	EGFR-TKI	NSCLC	In vivo*/*in vitro	Circ_0014235 confers Gefitinib resistance and malignant behaviors in non-small cell lung cancer resistant to Gefitinib by governing the miR-146b-5p/YAP/PD-L1 pathway	[202]
circRNA_102481	ROR1	NSCLC	In vitro	Tumor-derived exosomal circRNA_102481 contributes to EGFR-TKIs resistance via the miR-30a-5p/ROR1 axis in non-small cell lung cancer	[196]
hsa_circ_0109320	EGFR-TKI	NSCLC	Clinical Trial	Circular RNA profiling identified as a biomarker for predicting the efficacy of Gefitinib therapy for non-small cell lung cancer	[205]
circ-ERBB2	ERBB2	Lung cancer	In vivo*/*in vitro	Propofol suppresses lung cancer tumorigenesis by modulating the circ-ERBB2/miR-7-5p/FOXM1 axis	[204]
circFAM53B	c-met	Glioma	In vivo*/*in vitro	CircFAM53B promotes the proliferation and metastasis of glioma through activating the c-MET/PI3K/AKT pathway via sponging miR-532-3p	[190]
Circ_0001588	EPHB4	Glioma	In vivo*/*in vitro	. Circ_0001588 Upregulates ERBB4 to Promote Glioma Malignant Progression Through Sponging miR-1281	[191]
circ_0001162	EPHB4	Glioma	In vivo*/*in vitro	circ_0001162 promotes cell proliferation and invasion of glioma via the miR-936/ERBB4 axis	[192]
hsa_circ_0081519	EPHB4	Glioma	In vivo	. CircRNA EPHB4 modulates stem properties and proliferation of gliomas via sponging miR-637 and up-regulating SOX10	[193]
Circ_0044520	ROR2	Laryngeal squamous cell carcinoma	In vivo	. Circ_0044520 regulates the progression of laryngeal squamous cell carcinoma via the miR-338-3p/ROR2 axis	[194]
circ_0000745	ERBB4	Ovarian cancer	In vivo	. RNA-binding protein IGF2BP2 enhances circ_0000745 abundancy and promotes aggressiveness and stemness of ovarian cancer cells	[199]
hsa_circ_0006470	ROR1	Gastric cancer	In vitro	circRNA_0006470 promotes the proliferation and migration of gastric cancer cells by functioning as a sponge of miR-27b-3p	[195]
circ_0079558	MET	PTC	In vivo	Circ_0079558 promotes papillary thyroid cancer progression by binding to miR-26b-5p to activate MET/AKT signaling	[198]
circ-NSD2	DDR1	Colorectal cancer	In vivo*/*in vitro	NSD2 circular RNA promotes metastasis of colorectal cancer by targeting miR-199b-5p-mediated DDR1 and JAG1 signaling	[197]
circ-LAMP1	DDR2	T-cell Lymphoblastic Lymphoma	In vitro	Circ-LAMP1 promotes T-cell lymphoblastic lymphoma progression via acting as a ceRNA for miR-615-5p to regulate DDR2 expression	[200]

## Future prospect

**Fig. 5:**
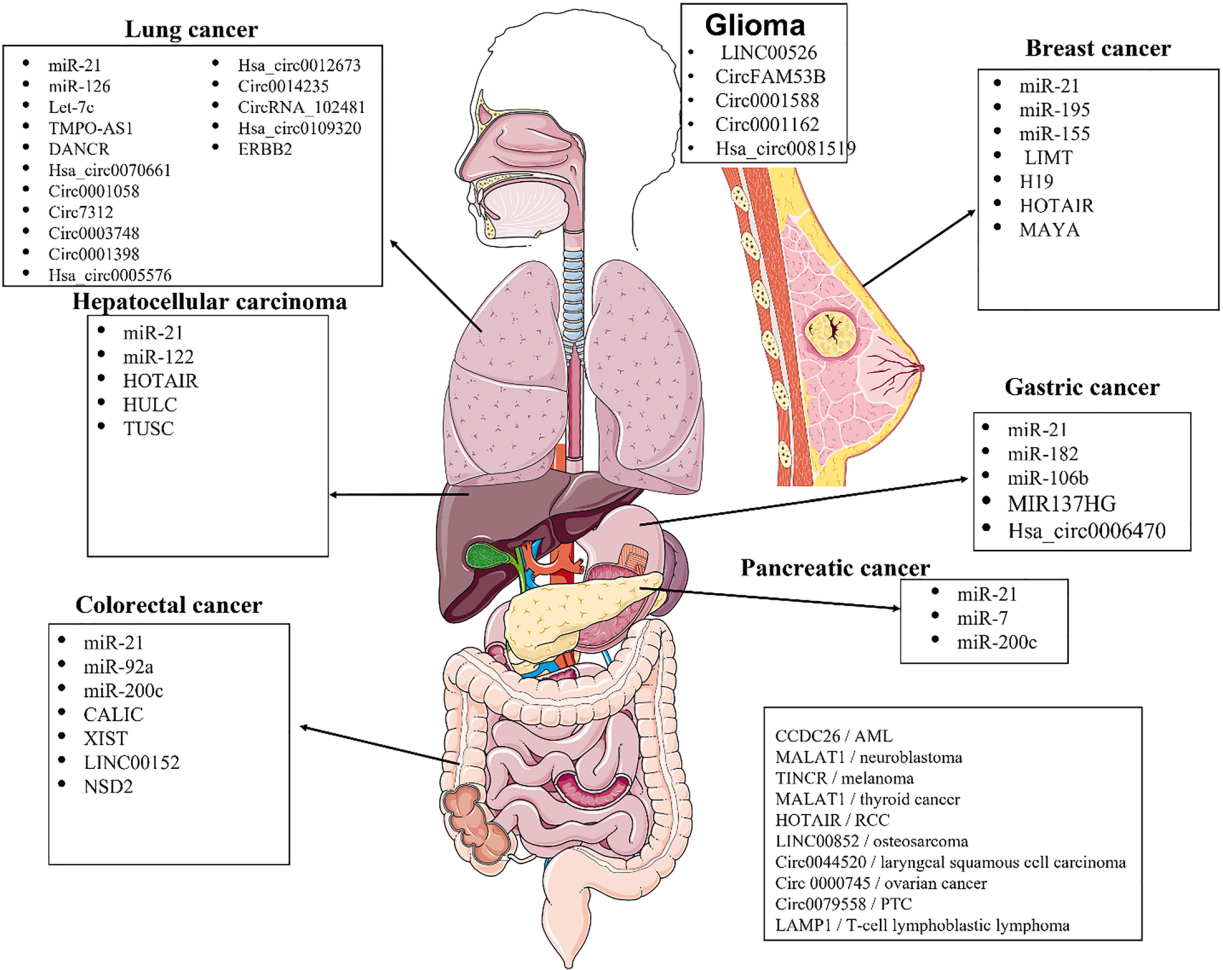
An illustration of RTK-associated ncRNAs with therapeutic application in solid tumors. This figure was created using the Servier Medical Art Commons Attribution 3.0 Unported License (http://smart.servier.com (accessed 10 July 2023))

Recent advances in the field of ncRNA research have revealed the critical role of ncRNAs in the regulation of RTK signaling in solid tumors. Non-coding RNAs have shown great potential as therapeutic targets for the treatment of RTK-associated solid tumors. However, further research is needed to fully understand the intricate mechanisms underlying ncRNA-mediated regulation of RTK signaling and to identify promising therapeutic targets (Fig. 5).

One promising avenue for future research is the development of ncRNA-based therapies, such as miRNA mimics, miRNA inhibitors, and circRNA-based therapeutics. These therapies have shown promising results in preclinical studies and hold great potential for the treatment of solid tumors. Several ncRNA-based therapies have already been developed for the treatment of various diseases, including cancer. For example, the miRNA mimic, miR-34a, is currently being evaluated in clinical trials for the treatment of various solid tumors. Another miRNA mimic, miR-16, has been shown to inhibit the growth of solid tumors in preclinical studies. Additionally, the combination of ncRNA-based therapies with existing conventional therapies, such as chemotherapy and RTK inhibitors, may increase treatment efficacy and reduce resistance.

Another area of future research is the identification of novel ncRNAs involved in RTK signaling pathways. Recent advances in high-throughput sequencing technologies and bioinformatics have enabled the identification of previously unknown ncRNAs and their potential roles in disease pathogenesis. Targeting these novel ncRNAs may provide alternative therapeutic strategies for the treatment of RTK-associated solid tumors.

Furthermore, the development of reliable methods for the delivery of ncRNA-based therapeutics to target tissues remains a challenge. Various delivery methods, such as nanoparticles, liposomes, and exosomes, have been explored for the effective delivery of ncRNA-based therapeutics. However, further optimization is needed to improve their specificity, stability, and efficacy.

ncRNAs can also be used as biomarkers for the diagnosis and prognosis of solid tumors. Several studies have shown that the expression levels of certain ncRNAs are altered in solid tumors compared to normal tissues. For example, the expression of miR-21 is upregulated in various solid tumors and is associated with poor prognosis. The expression of miR-195 is downregulated in various solid tumors and is associated with a better prognosis.

Several methods are currently available to experimentally identify ncRNAs from samples, including enzymatic/chemical RNA sequencing, use of cDNA libraries, microarray analysis, and genomic SELEX. The RNA sequencing methods though not impeded by secondary/tertiary RNA structures (in contrast to cDNA-based methods), have some limitations including the inability to discriminate between RNAs with similar size, and limited size of identified RNAs, and it is limited to highly abundant RNAs. On the other hand, microarray analysis can simultaneously detect multiple RNAs and have great potential. Genomic SELEX methods are based on the generation of RNAs from genomic DNAs in vitro and can be used to identify RNAs that are not necessarily expressed [208].

Furthermore, the development of new technologies, such as single-cell sequencing and CRISPR/Cas9 genome editing, will allow for a more comprehensive understanding of the role of ncRNAs in the regulation of RTK signaling in solid tumors. Single-cell sequencing will enable the identification of ncRNAs that are specifically expressed in tumor cells, which can be targeted for therapy. CRISPR/Cas9 genome editing will allow for the precise manipulation of ncRNA expression levels, which can be used to study the function of ncRNAs in solid tumors.

## Conclusion

Non-coding RNAs have emerged as promising therapeutic targets for the treatment of RTK-associated solid tumors. MicroRNAs, circular RNAs, and long ncRNAs have been extensively studied in the context of RTK signaling, and their dysregulation has been implicated in the development and progression of various solid tumors. Future research aims to develop ncRNA-based therapies, identify novel ncRNAs involved in RTK signaling pathways, use of ncRNAs as biomarkers for diagnosis and prognosis, and optimize delivery methods for these therapeutics. These efforts will hold great potential and advance our understanding of the role of ncRNAs in solid tumors, as well as lead to the development of more effective and personalized treatments for patients with solid tumors.

## Data Availability

As this is a review article, the data and material analyzed were obtained from previously published sources, and as such, all data analyzed are publicly available. Details of the sources of information used in this review are provided in the reference list. Additionally, any specific materials referenced in this review are obtainable through their respective sources, as cited in the text.

## Abbreviations

pri-miRNA: Primary miRNA
miRISC: MiRNA-induced silencing complex
AGO: Argonaute
EMT: Epithelial mesenchymal transition
AUC: Area under the curve
RON: Receptor d’Origine Natais
Pri-lncRNA: Primary long non-coding RNA
ADAM17: A Disintegrin and Metalloproteinase 17
PTEN: Phosphatase and Tensin Homolog
P53: Tumor Protein P53
RAS: Rat sarcoma
BCL2: B-Cell Lymphoma 2
MET: Mesenchymal-Epithelial Transition Factor
PRDX2: Peroxiredoxin 2
DNMT3B: DNA (Cytosine-5)-Methyltransferase 3 Beta
CDK6: Cyclin-Dependent Kinase 6
MCL1: Myeloid Cell Leukemia 1
EGFR: Epidermal Growth Factor Receptor
HDAC4: Histone Deacetylase 4
Notch: Neurogenic locus notch homolog protein
c-MYC: Myelocytomatosis Proto-Oncogene Protein
MAPK: Mitogen-Activated Protein Kinase
PI3K/AKT: Phosphoinositide 3-Kinase/Protein Kinase B
NSCLC: Non-Small Cell Lung Cancer
NOV340: A type of liposome
let-7b: MicroRNA let-7b
MRX34: A therapeutic approach with miR-34 mimics in liposomes
DOTMA: *N*-[1-(2,3-Dioleoyloxy)propyl]-*N*,*N*,*N*-trimethylammonium
SiO2NPs: Silica Nanoparticles
HER-2: Human Epidermal Growth Factor Receptor 2
HDAC4: Histone Deacetylase 4
HA: Hyaluronic acid
PEI: Polyethyleneimine
GAPB/NRF2: Glyceraldehyde-3-Phosphate Dehydrogenase Binding Protein/Nuclear factor erythroid 2-related factor 2
SESN1: Sestrin 1
CDK4: Cyclin-dependent kinase 4
lncRNAs: Long noncoding RNAs
HCC: Hepatocellular carcinoma
SCLC: Small cell lung cancer
RTK: Receptor tyrosine kinase
miR: MicroRNA
RCC: Renal cell carcinoma
circRNA: Circular RNA
HIF1A: Hypoxia-inducible factor 1-alpha
ROR1: RTK-like orphan receptor 1
GAS5: Growth arrest-specific 5
EIciRNAs: Exon–intron circRNAs
TF: Transcription factor
XIST: X-inactive specific transcript
AXL: AXL receptor tyrosine kinase
Linc00152: Long intergenic non-protein coding RNA 152
CALIC: Colon cancer-associated lncRNA in CTCs
MALAT1: Metastasis-associated lung adenocarcinoma transcript 1
MAYA: Metastasis-associated Y-chromosome lncRNA
SPRY4-IT1: SPRY4 intronic transcript 1
FGFR2: Fibroblast growth factor receptor 2
GAS5: Growth arrest-specific 5
HIF1A-AS2: HIF1A antisense RNA 2
VEGFA: Vascular endothelial growth factor A
AR: Androgen receptor
MMP-13: Matrix metalloproteinase-13
K-RAS: Kirsten rat sarcoma viral oncogene homolog
P-ERK: Phosphorylated extracellular signal-regulated kinase
UCA1: Urothelial carcinoma-associated 1
CRNDE: Colorectal neoplasia differentially expressed
PCAT1: Prostate cancer-associated transcript 1
TEK: Tyrosine Kinase
ERBB4: Erythroblastic Leukemia Viral Oncogene Homolog 4
EPHB4: Ephrin Type-B Receptor 4
SOX10: SRY (Sex Determining Region Y)-Box 10
DDR1: Discoidin Domain Receptor Tyrosine Kinase 1
JAG1: Jagged 1
IGF2BP2: Insulin-like growth factor 2 mRNA binding protein 2
EGFR-TKIs: Epidermal growth factor receptor tyrosine kinase inhibitors
NSD2: Nuclear Receptor Binding SET Domain Protein 2
MET: Mesenchymal-Epithelial Transition
AKT: Protein Kinase B
MAPK1: Mitogen-Activated Protein Kinase 1
PD-L1: Programmed Death-Ligand 1
Gefitinib: Epidermal Growth Factor Receptor Tyrosine Kinase Inhibitor
YAP: Yes-Associated Protein
circ-ERBB2: Circular RNA derived from ERBB2 gene
FOXM1: Forkhead Box Protein M1
ASK1: Apoptosis signal-regulating kinase 1
circASK1: Circular RNA derived from ASK1 gene
tsRNA: Transfer RNA-derived small RNA
piRNA: PIWI-interacting RNA
